# Geoelectric characterisation of the junction of seismically active Delhi Hardwar Ridge and Delhi Sargodha Ridge

**DOI:** 10.1038/s41598-023-42722-w

**Published:** 2023-10-28

**Authors:** Gautam Rawat, Kapil Mohan, S. Dhamodharan, Harendra Dadhich, Prasanta Chingtham, Kalachand Sain, O. P. Mishra

**Affiliations:** 1 Wadia Institute of Himalayan Geology, 33 GMS Road, Dehradun, 248001 India; 2 National Center for Seismology, Ministry of Earth Sciences, Lodhi Road, New Delhi, 110003 India

**Keywords:** Solid Earth sciences, Geophysics, Seismology, Tectonics

## Abstract

A magnetotelluric (MT) geophysical survey for the first time has been conducted for the geoelectric characterization of the junction of the contact zone of NNE-SSW striking Delhi Hardwar Ridge (DHR) and NW–SE trending Delhi Sargodha Ridge (DSR) in the Rohtak area, Haryana which has experienced 15 earthquakes of M2.0–M4.4 from April to August 2020. A total of 08 MT sites are acquired along a NW–SE profile of length 50 km. From the 2D MT data inversion, the DHR and DSR are for the first time characterized by equal values of moderate resistivity of 100 Ohm m at two depths. The resistivity variation for DHR corresponds to 100 Ohm m from the surface to the depth of 20 km, whilst DSR is found associated with the same value of resistivity extending in the NW direction. The DHR has been found striking NE-SW with a very shallow central axis (less than 400 m) having a width of 12–15 km forming half grabens on both limbs supported by shallow faults. The DSR has been found bifurcated from DHR at a depth of 12–13 km and extended in the NW direction. The DSR has been generated due to flexure bulging caused by collision and anticlockwise rotation of the Indian plate in the Eocene period. A NE striking steep dipping reverse fault (F1) has also been identified about 15 km west of the DHR. It is inferred that the DSR got upthrusted along this fault and became shallower in the NW region. The seismicity in the Rohtak and surroundings is located at the bifurcation points of DHR and DSR and the contact zone of DSR and reverse fault F1. The reverse fault F1 is also active and has generated microseismicity in the past.

## Introduction

**Figure 1:**
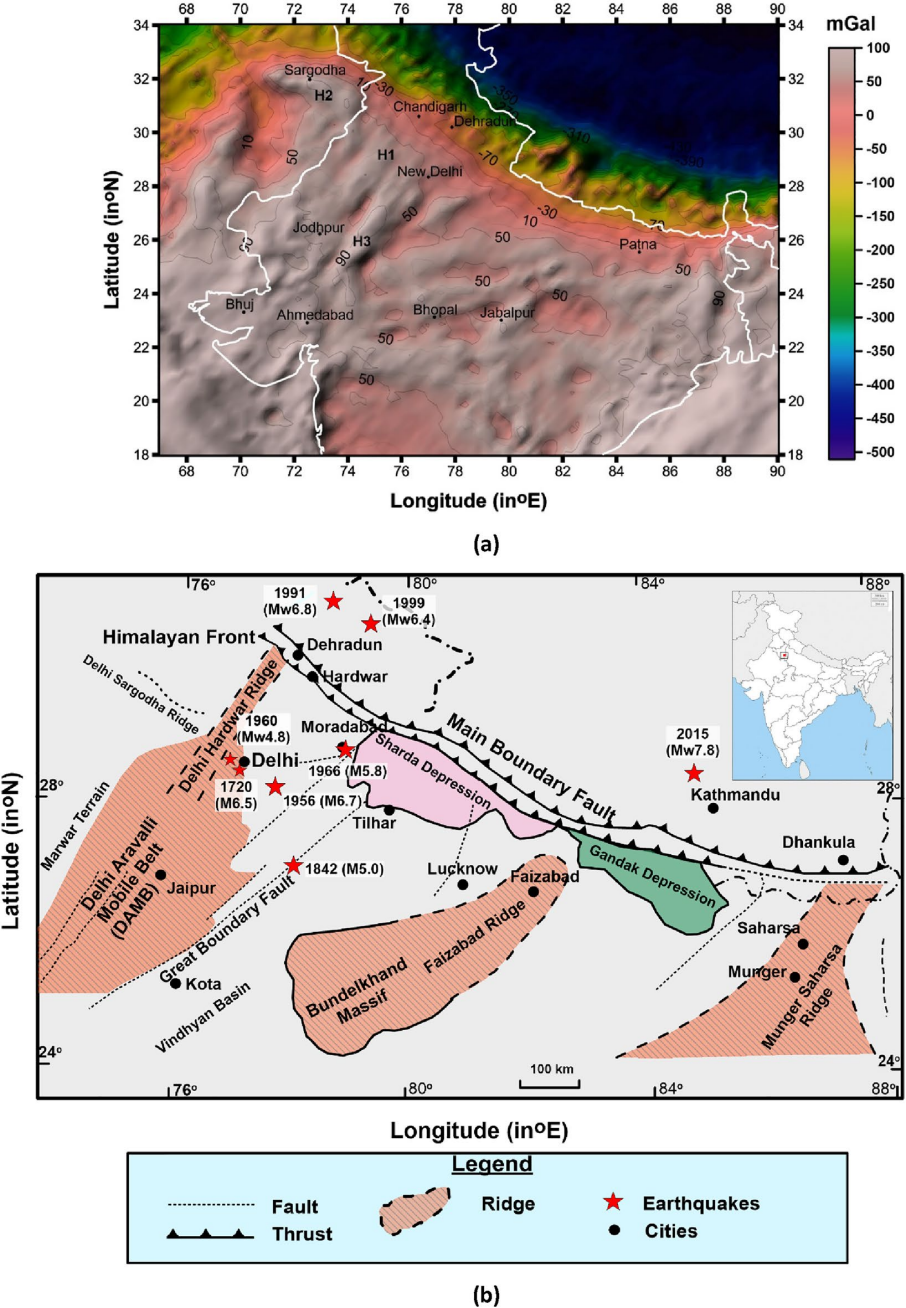
(**a**) Bouger gravity map of northern & central India and surroundings (after WGM2012 Model from https://bgi.obs-mip.fr/data-products/grids-and-models/wgm2012-global-model/) with marked prominent gravity highs (H1, H2 and H3). The map is created with the software ArcGIS version 10.7.1. (https://www.esri.com/en-us/arcgis/products/arcgis-desktop/resources). (**b**) the basement structure of the Ganga Basin based on Fuloria^3^ and Sastri et al.^4^, the location of Delhi Sargodha Ridge is plotted after GSI^5^. The figure is digitized using the software ArcGIS version 10.7.1. (https://www.esri.com/en-us/arcgis/products/arcgis-desktop/resources).

India’s National Capital Region (NCR) is a densely populated habitat and hub for national activities. It falls under Zone III (third highest seismic zone) and Zone IV (2nd highest seismic zone) in the seismic zoning map of India^1^. It has frequently been experiencing shaking from earthquakes originating in the Himalayas due to far-field sources and also due to the activation of local sources. In the past, the Delhi region had experienced far-field earthquakes including the 1905 Kangra earthquake of magnitude 7.8, the 1991 Uttarkashi earthquake of magnitude 6.8, the 1999 Chamoli earthquake of magnitude Mw 6.6, and the 2015 Gorkha earthquake of magnitude 7.8 (Fig. 1). The Delhi region had also been affected by nearby earthquakes like the 1720 Delhi earthquake of magnitude M 6.5, the 1956 Bulandshahar earthquake of magnitude M 6.7 and the 1960 Gurgaon earthquake of magnitude Mw 4.8. An earthquake of magnitude M4.0 also occurred on 28 July 1994 and caused damage to the minaret of the Jumma Masjid of Delhi^2^.

A Bouguer anomaly map for Northern India including the Ganga Basin was proposed by Ravi Kumar et al.^6^ in which they suggested that the interaction of the Delhi Aravalli Mobile Belt (DAMB) and its boundary with the Delhi–Sargodha faults Ridge (DSR) (a gravity high) and to the Himalayan front in the north while extending towards the Western Himalayan front via Delhi causes seismic activity. Similar observations are also found in the Earth Gravity Model (EGM), 2012 map (Fig. 1). Based on the Earth Gravitational Model (EGM, 2008), the Delhi Hardwar Ridge (DHR) was proposed as a gravity high, a horst having steeply dipping normal faults on both sides that continues north-eastward to the Karakoram fault across the Main Frontal Thrust (MFT)^7^. Based on a 3D inversion of gravity data, Dwivedi et al.^8^ anticipated that the NE trending Delhi Fold Belt ricocheted westward towards the shallower DSR due to the anti-clockwise rotation and NW corner depression in the Indian plate after collision with the Eurasian plate post (in the post-Eocene).

Richter^9^ studied the seismotectonic of Delhi and its surroundings and suggested block faulting. Based on the seismicity between 1962 and 1972, Chouhan^10^ proposed that the juncture of the DSR, the DHR, and the axis of the Delhi Fold Belt are the most active regions.

Computing the focal mechanism of the 14 earthquakes that occurred during 2001–2004 in the Delhi region, Shukla et al.^11^, anticipated the association of these earthquakes with two major structures (i) the Mahendragarh–Dehradun Fault (MDF) trending NNE–SSW and (ii) the Delhi–Sargodha Ridge (DSR), trending NW–SE. Rohtak was suggested as the seismically most active region.

Bansal et al.^12^, through seismological investigation, have suggested that NE-SW trending DHR splits the two structural environments in Delhi: reverse faulting in the west and normal faulting in the east. West of Delhi, the WNW-ESE striking DSR intersects MDF. Seismically, the region between DHR/MDF and DSR has been extremely active over the last twenty years.

The Rohtak is situated ~ 70 km southwest of Delhi and is a district in Haryana state located at the proposed junction of DHR and DSR. In the past, Rohtak was also affected by swarm activity during 1963–1965^10,13^. Since 2000, a total of 471 earthquakes of 1.2 ≤ M ≤ 4.9 were recorded in the 50 km vicinity of Rohtak, including 05 earthquakes of M4–M4.9. During the last two decades, in the Delhi-NCR region, an earthquake with a maximum magnitude of M4.9 occurred in the Rohtak area. DHR and DSR weren’t characterized earlier in the Rohtak area, which is considered to be the probable location of the contact zone between the two ridges (DSR and DHR) because of high levels of anthropogenic noise. Looking into the importance of this seismically active, structurally complex part of NCR, a Magnetotelluric survey is planned to characterize both DHR and DSR as well as the characterization of seismicity of the region.

## Geology and tectonics

The NCT Delhi falls on the western edge of the Ganga basin, east of the DHR, surrounded by the Himalayas in the north and the Aravalli ranges in the south (Fig. 1b). The Delhi Aravallis Fold Belt (DAFB) is a 700 km long NE-SW trending hill range formed due to collision of Marwar Craton present in the NW and Bundelkhand Craton present in the SE during Proterozoic^14^. The Aravalli Fold Belt belongs to the Lower to Middle Proterozoic whereas the Delhi Fold Belt formed during the Middle to Upper Proterozoic. The DHR was suggested as the northern extension of the Delhi Fold Belt and considered as covered under Tertiary sediments while extending towards the Himalayas in the north. The western limit of the Ganga Basin is controlled by DHR. The MDF is an NE–SW striking, 295 km-long fault that passes along the eastern margin of the DHR and connects the Himalayan Frontal Thrust (HFT) in the north to the Indian peninsular craton in the south^15^. The DSR is another important feature that is considered a flexural bulge on the foreland basin of Himalaya^16^ and is bordered by the Sahaspur and Bikaner Basins^11^ to the north and southwest, respectively. The other tectonic features of the Delhi region are, the Sohna fault and Mathura fault in the south, the Great Boundary fault in the SE, and Moradabad fault in the east. The Main Boundary Thrust (MBT) and Main Central Thrust (MCT) are present in the north of Delhi.

Interestingly, Delhi's rocks date from two distinct geological periods: the Precambrian and the Quaternary^17,18^. The intervening annals are missing as the area has been exposed to erosion since late Pre-Cambrian times after rising beneath the sea. Naha et al.^19^ and Ahmad and Ahmad^20^ posit that these Pre-Cambrian rocks are from the Alwar Series of the Delhi system. The area consists of 95% Quaternary sediments, while the remaining 5% consists of Meso to Neo-Proterozoic rocks of the Delhi Supergroup which are present in the south and south-west, intruded by more than one phase of acidic and basic intrusives of Neo-Proterozoic age (Post Delhi Intrusives) and the Tertiary rocks in the north-east. Delhi Supergroup includes the metasedimentary rocks of Alwar and Ajabgarh Group.

The Delhi area is mainly covered with Quarternary to recent sediments (of ≤ 1.65 Ma) present in the unconsolidated stage above the quartzite of the Alwar series of the Delhi Supergroup. These Quaternary sediments are comprised of newer Alluvium (consisting of clay, silt, and sand present mainly in the active and older flood plains of the River Yamuna) and the older Alluvium (consisting of clay with minor lenticular fine sand, silt, and Kankar beds). A variable sediment thickness is observed in the east and west sides of DHR, having more towards the west (about 290 m).

Three types of folding were reported in the rocks of the Delhi region by Gangopadhyay and Sen^21^. Later on Naha et al.^22^ and Roy^23^ also confirmed numerous foldings and various phases of metamorphism over time in the Delhi area. Out of the proposed three generations of folds, the axis of first-generation folds reported trending similar to the main ridge i.e. NNE-SSW; the second-generation folds are observed trending in the NE-SW direction (in the Mehrauli- Tuglakabad area) and the third-generation folds are observed at Anand Parbat area trending in the NW–SE direction. In the highly jointed rocks of the Delhi region, the conjugate vertical to sub-vertical joints (trending NNE-SSW and WNW- ESE) were reported by Kachroo and Bagchi^24^. The NNE-SSW, NE-SW, and WNW-ESE trending faults were also inferred in the Delhi area by Srivastava et al.^25^.

## Magnetotelluric inversion and interpretation

**Figure 2:**
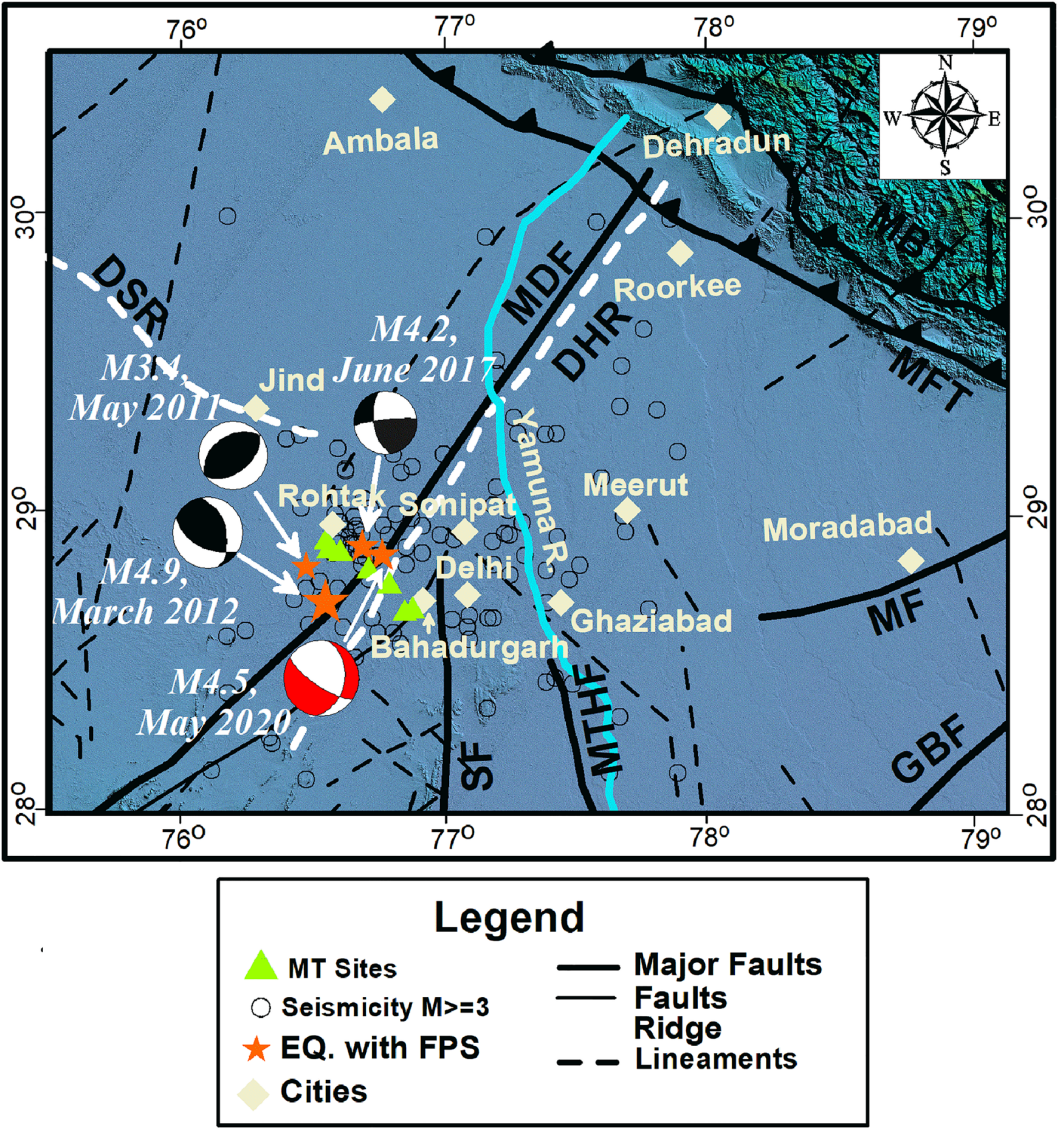
The locations of MT sites (shown with yellow triangles) acquired in the study area overlapped on a seismotectonic atlas of India and its Environs (after GSI^5^). The beach balls are from the fault plane solutions of the significant past earthquakes in the area. The red beach ball is of the latest earthquake (May 2020) of M4.5 that occurred in the study area overlapped on Shuttle Radar Topography Mission data of 90 m resolution (http://srtm.csi.cgiar.org). The map is prepared using the software ArcGIS version 10.7.1. (https://www.esri.com/en-us/arcgis/products/arcgis-desktop/resources). MDF: Mahendragarh Dehradun Fault; DHR: Delhi Hardwar Ridge; DSR: Delhi Sargodha Ridge; SF: Sohna Fault; MTHF: Mathura Fault; MF: Moradabad Fault; GBF: Great Boundary Fault; MFT: Main Frontal Thrust and MBT: Main Boundary Thrust.

**Figure 3:**
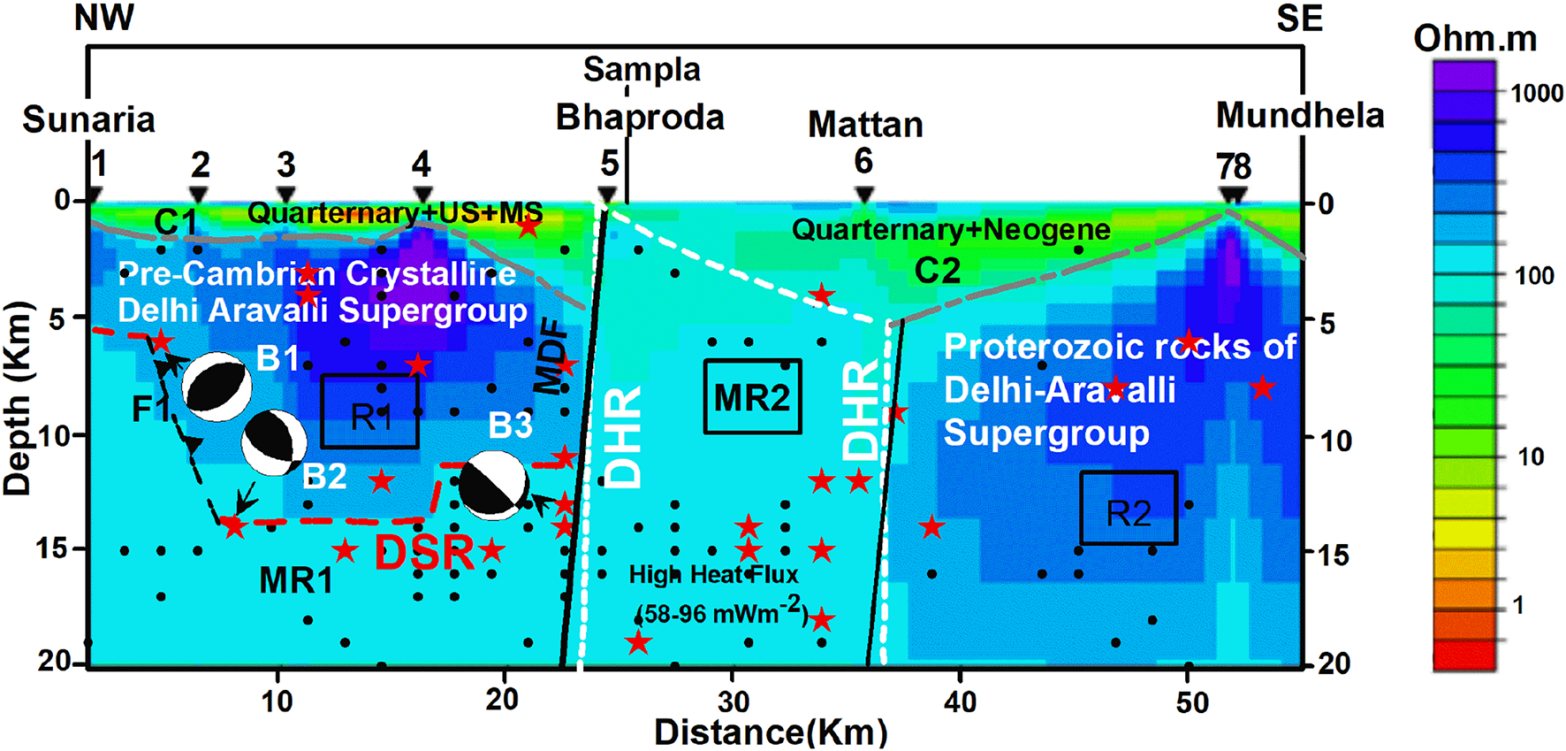
The subsurface resistivity section (TM mode) of the Rohtak region overlapped with the seismicity of magnitude 2.0 ≤ M ≤ 2.9 (shown with black dots) occurred between latitude 28°N–29.5°N and longitude 76°E–77°E from January 2000 to April 2022 taken from https://seismo.gov.in/ managed by National Center for Seismology, Government of India. The bigger stars are seismicity of magnitude 3.0 ≤ M ≤ 5.1. DSR: Delhi Sargodha Ridge; DHR: Delhi Hardwar Ridge; MR1: Medium Resistor 1; MDF: Mahendragarh Dehradun Fault; MR2: Medium Resistor 2; R1: Resistor 1; R2: Resistor R2. The beach ball (B1) suggests a NE striking steep dipping reverse fault motion; the beach ball (B2) suggests a NW striking reverse with strike-slip fault motion and the beach ball (B3) suggests an NNE striking, normal with strike-slip fault motion.

A profile of 8 Magnetotelluric sites has been acquired in the NW- SE direction initiating from Sunaria village (Rohtak, Haryana) in the NW to Mundhela village, Delhi in the SE covering a length of 50 km (Fig. 2). The profile was acquired in the vicinity of the densely populated, tectonically complex and seismically very active Rohtak-Delhi area located in the SW part of the National Capital Region (NCR) due to an increase of the seismicity in the area during April-August 2020. By exploiting the 2D MT data inversion, the resistivity depth sections for transverse magnetic (TM), transverse electric (TE), and TE + TM modes to a depth of 20 km have been prepared and shown as Fig. 3, Supplementary Figs. 3 and 4, respectively. It is quite difficult to get clean (affected by a very low cultural noise) MT sites in the region. As site 7 and site 8 are very close, an effort has also been made to invert the data removing site 8. The TM mode section thus produced is given as supplementary Fig. 5. A significant difference is not observed even after removing site 8. Therefore, site 8 is included in the final model. The geoelectric depth sections produced in the current study (Fig. 3) suggested a resistivity of 1–2000 ohm m in the study area.

Based on estimated resistivity, the study region can be divided into three major parts; one in the NW of the profile from village Sunaria to Bhaproda, having a length of ~ 20 km (NW region), and the second in the SE part of the profile from village Mattan to Mundhela, with a length of ~ 20 km (SE region) and the third, a central part of about 12–15 km in length between villages Mattan and Bhaproda. Depth-wise, the NW region can further be sub-divided into three sub-parts; a conductive layer (C1), with a resistivity value ranging from 1 to 30 Ohm m (Fig. 3) having a thickness of 1–2 km from the surface; a central resistive layer (R1) (with resistivity ranging from 300 to 2000 Ohm m) having a thickness of ~ 10 km followed by a third layer (MR1) with moderate resistivity of the order of 100 Ohm m. The study area falls at the junction of the Punjab plains (in the NW) and Ganga Basin (in the SE), separated by the Aravallis in the central part. As per the stratigraphy sequence proposed by GSI^26^ (Table 1), the Punjab plains are covered by Siwaliks with Quaternary as the top layer. In the eastern part of the Punjab plains, the recent alluvium is present in the northern areas, close to the Himalayas; however, the southern part is covered by dune-type Quaternary sediments^27,28^. The stratigraphic sequence proposed for the Rohtak district (Table 2) suggests the presence of Quaternary sediments (newer and older Alluvium (of Pleistocene/Upper Siwalik both)) followed by Proterozoic rocks of the Delhi supergroup.

**Table 1:** The generalized stratigraphy of Punjab Basin^26^.

Type	Age	Thickness (in m)
Upper Siwalik	Pliocene	450–2300
Middle Siwalik	Upper Miocene	1000–2850
Lower Siwalik	Middle Miocene	1250–2200
Dharamsala/Murree	Lower Miocene	1750–4600
Subathu	Eocene	100–800
Unconformity		
Riasi Limestone and Equivalent? Proterozoic/Lower Mesozoic		
Unconformity		
Crystalline basement

**Table 2:** Generalised stratigraphic sequence of rocks in Rohtak district^29^.

System	Geological age	Stratigraphic unit
Quaternary	Recent	Wind-blown sand
		Newer alluvium
	Pleistocene	Older alluvium
Unconformity		
Proterozoic	Quartzites and other Associated rocks of Delhi Supergroup	

Therefore, the first layer (C1) of the NW region of the study area represents the Quarternary sediments (newer and older Alluvium (of Pleistocene/Upper and Middle Siwalik), and the second Layer (R2) represents the Proterozoic rocks of the Delhi supergroup. Moving from NW to SE along the acquired MT profile (from site number 1 towards site number 5), the sediment thickness is found to increase from ~ 500 m to ~ 3 km with basement undulation indicating the presence of unconformity. The top 400–500 m layer having a resistivity of 1–5 Ohm m is inferred as the recent Alluvium/Quarternary sediments followed by the Siwaliks of thickness about 500 m below site 1 and ~ 3 km below site 5.

## Discussion

The Oil and Natural Gas Commission (ONGC) in 1968 identified the three ridges (DHR in the west, Faizabad ridge in the center, and Munger Saharsa ridge in the east) in the Indo Gangetic plains based on drilling data and geophysical surveys (gravity, aeromagnetic and seismic). Later Valdiya^30^ correlated these basement ridges with the transverse structures present in the Himalayas. The moderately resistive feature MR2 (Fig. 3) has a resistivity of ~ 100 Ohm m. It's in the central part of the profile, coinciding with the location of the DHR. It might be the DHR. A distinctive resistivity contrast is present between the feature R1/MR1 and MR2 coinciding with the location of Mahendragarh Dehradun Fault (MDF), inferred as MDF passing in the close vicinity/below the Bhaproda village.

The SE region of the study area, located between the villages Mattan and Mundhela (Fig. 3), can be divided into two major sub-parts along the depth of the profile; a top layer (C2) having a resistivity of about 10–50 Ohm m, thinning towards SE part of the profile (a thickness of ~ 4 km in the central portion of the profile below site 6 to a thickness of ~ 500 m in the SE part of the profile below sites 7 and 8) followed by a resistive layer (R2) having a resistivity of 300–1000 Ohm m. The southernmost site of the profile is 5 km NE of Najafgarh (Delhi), where a bedrock depth of approximately 300m was suggested by Shekhar^31^ below alluvium^24^. The DHR is the western boundary of the Ganga Basin. According to Sastri et al.^4^, the DHR represents a north–north-eastward extension of the Delhi Aravalli fold belts which cannot be delineated much yonder to Meerut, and the trend is possibly covered by a Neogene cover. Therefore, the conducting layer C2 might consist of Neogene and Quarternary sediments, and the thickness of Neogene sediments decreases towards the SE direction. It is, therefore, inferred that the alluvium is found only above the bedrock in the Delhi Region. The DHR delimits the western limit of the Ganga Basin, where the oldest sedimentary sequence, the Vindhyans, may gradually thin out. However, on the tectonic map of the Ganga Basin prepared by ONGC in 1968, the Moradabad fault limits these sediments to the west. The Proterozoic rocks consisting of the Delhi and Aravalli Supergroups are suggested in the western part of the Ganga Basin, north, and west of the Great Boundary Fault^32^. The Delhi area is mainly covered with quartzite from the Delhi Supergroup that is interbedded with micaceous schist bands. Srivastava et al.^25^ later named it as Alwar formation of the Delhi Supergroup, while Kachroo and Bagchi^24^ classified them as the Barkhol formation of the Ajabgarh Group of the Delhi Supergroup. The Alwar series comprises mainly quartzite, with garnetiferous schist, graphitic, subordinate carbonaceous, and ash beds, whereas the Ajabgarh series consists dominantly of limestone, quartzite, slate, and phyllite^28^. The layer R2, underlying layer C2, might be the Proterozoic rocks of the Delhi Supergroup. The sediment thickness/basement depth in the SE region is found to decrease from site 6 (~ 4 km) in the central part of the profile to sites 7 & 8 (about 1 km) in the SE part. The undulation of about 3km and sharp change in resistivity (50–1000 Ohm m) suggests the presence of unconformity dipping towards NW (Fig. 3). A sharp contrast of resistivity is also found between feature MR2 (~ 100 Ohm m) and R2 (200–1000 Ohm m) which indicate the presence of a subsurface fault below Mattan village and named as Mattan Fault.

The Bedrock at Hasangarh, Sampla block of Rohtak, encountered at a depth of 370.0 m^33^, located at the central part of the DHR. A total of 11 exploratory boreholes were drilled by CGWB in the old Rohtak district (covering the area of Jhajjar also), out of which the bedrock was encountered in 7 boreholes from 155 m near Bahu to 315.5 m near Jhajjar^29^. The Sampla village is located at the central axis of DHR, close to site 5 (Bhaproda village). Therefore, the depth of the MR2 can be inferred as ~ 400 m at the top of the ridge.

The DAFB with a Proterozoic origin has a very noticeable exposure in the form of a NE striking series of hills from Delhi to Mount Abu, Rajasthan. During subduction and/or collision, the NW Indian Shield's Mesoproterozoic Delhi and Paleoproterozoic Aravalli fold belts were most likely formed due to plate tectonic processes^34^. The DAFB is overlain by the alluvial sediments in Delhi and is assumed to extend further in the NNE direction as the DHR^35^. Sastri et al.^4^ have pointed out that DHR was not delineated by the seismic survey beyond the Meerut area. They further suggested that DHR has the smallest area extent (6000 sq km) among all three ridges. The shallow character of the DHR has also been described by Karunakaran and Ranga Rao^36^. Arora et al.^37^ and Arora and Mahashabde^38^ have described DHR as a 15 km wide electrical conductor with a resistivity of the order of ~ 2 Ohm m located at a depth of 15 km trending in 60°E (of Aravalli range) and enters into the Himalaya. In the present study, the top axis of DHR has been inferred at a depth of ~ 400 m in the study region with a resistivity value of 100 Ohm m and a width of ~ 15 km. Arora et al.^39^ have anticipated the segmentation of NW Himalaya (controlled by the rifts, nappes, and subsurface ridges) along its strike. Gahalaut and Kundu^40^ have suggested the ridges, including DHR, as the rupture barriers. Based on the aeromagnetic as well as gravity surveys, the GSI^5^ has proposed a 295 km long fault, extending from HFT in the north to the Indian peninsular craton in the south along the DHR and named it as Mahendragarh Dehradun Fault (MDF). Based on Ground Penetrating Radar (GPR) investigations in its vicinity, Patel et al.^15^ have proposed it as a normal fault system at a shallower depth. Moreover, based on seismicity and geomorphological characteristics, they have proposed a normal and oblique-slip motion along MDF. The present study found a very sharp resistivity contrast at the suggested location of MDF, therefore, confirming it as a steep dipping fault.

Three major gravity investigations in the area have been conducted in the recent past and have proposed the following: (i) the Aravalli Delhi Mobile Belt (ADMB) and its margin faults extend to the Western Himalayan front via Delhi where it interacts with the Delhi–Lahore/Sargodha Ridge (DSR) (a gravity high) and further north with the Himalayan front causing seismic activity^6^, (ii) the Delhi–Hardwar trend (a gravity high and a horst with steeply-dipping normal faults on either side) continues in the north-eastward across the surface trace of the Main Frontal Thrust to the Karakoram fault^7^ and (iii) the NE trending Delhi Fold Belt (a gravity High) deflected westward towards the DSR (a gravity High) due to NW corner indentation and anti-clockwise rotation of Indian plate (post-Eocene collision) and producing clustered micro-seismicity caused by high strain resulting from crustal buckling of Delhi Fold Belt and DSR^8^.

The feature MR1 is present in the NW region of the geoelectric depth section west of MR2 (Fig. 3) with an equal resistivity of ~ 100 Ohm m at a depth of about 12–13 km along a gravity high and coincides with the suggested location of DSR and is inferred as DSR. In the past, the DSR was proposed as (i) an NW–SE trending feature extending from Lahore/Sargodha in the NW to Delhi in the SE based on the alignment of outcrops of the Archean rocks (seen at Sargodha, Chiniot and Kirana hills) with the outcrops of the Aravalli rocks (seen at Tosham, Haryana)^41^ and (ii) as a linear trend of the Bouguer gravity high in NW India along the axis of the DSR^8^. It has no surface expression. According to Datta and Sastri^42^, the upliftment of the area north to DSR continued till the middle Tertiary, and only since Miocene, the ridge acquired its present shape. The MR1 and MR2 in the geoelectric depth section (Fig. 3) have shown similar resistivity (~ 100 Ohm m) which raises two questions: (i) The Delhi Aravalli Fold Belt (DAFB) drifted westward and continued as the DSR or (ii) DAFB and its margin faults extend to the Western Himalayan front via Delhi but get bifurcated in the Rohtak area and one branch extended as DSR in the NW and the other extended as DHR in the NE.

The gravity maps generated in past studies including the Bouger gravity map proposed by EGM, 2012 have proposed gravity highs marked as H1 and H2 in Fig. 1 at the proposed locations of DHR and DSR, respectively both implying a high-density material. The Delhi Aravalli Supergroup of rocks embraced metavolcanics and metasediments of the Proterozoic period, suggesting high gravity signatures. In India, the temperature measurements were carried out at 82 different locations drilling 220 boreholes^43^ with a total of 07 in the Aravalli Craton. The Proterozoic Aravalli Supergroup, present in the region is associated with high heat flux, varying from 56 to 96 mW m^−2^, with a mean value of 68 mWm^−2^^44–47^, which is higher than the heat flow values observed in other cratons (Bundelkhand Craton (32–41 mWm^−2^), Bastar Craton (51–63 mWm^−2^) Dharwar Craton (25–43 mWm^−2^) and Western Dharwar Craton (29–39 mWm^−2^)]^43^ and also the most of the other locations where temperature measurements were conducted. The highest value of 96 mW m^−2^ was estimated at Tosham, Haryana. In the absence of any recent tectonothermal event, the high heat flow in Delhi Aravalli Supergroup might be related to large numbers of felsic intrusives^43,48^ and large crustal-scale faults that may act as channels for the mantle heat flow as well as aiding the recent uplift. Mishra and Ravi Kumar^48^ have suggested that the presence of high conductivity and low-density material rocks in the upper mantle dipping away from the ADMB indicates the remanence of the subducted rocks that are metamorphosed to give rise to fluids causing low resistivities at shallow depths in the ADMB^48^. The presence of similar resistivity in the west (at MR1) at about a depth of 12–13 km suggests the formation of MDF along the western axis of DHR during the collision of the Indian and Eurasian Plates. Rao^41^ suggested that both of the ridges (DHR and DSR) are concealed under the alluvium of the plains while extending from Delhi to Dehradun and Delhi to Lahore. Commenting on the focal mechanism proposed by Shukla et al.^11^ of the Delhi earthquake, Dubey et al.^49^ suggested that the earthquakes that occurred in the NW /Rohtak area and have a different orientation than MDF might have been caused by the lithospheric crustal loading of the Himalaya orogen on the DSR^49^.

The GSI^5^ in the proposed seismotectonic map has suggested a lineament in the west to DHR/MDF (Fig. 1). In the geoelectric depth section (Fig. 3) generated in the present study, a fault (F1) has also been identified between site1 and site 2 where MR2/DSR has stepped up in the NW direction on extending this lineament in the south. Bansal et al.^12^ and Shukla et al.^11^ have proposed the focal mechanism of the earthquake in the vicinity of this fault. The focal mechanism suggested it as a NE striking steep dipping reverse fault (Fig. 3) which implies that the DSR, which was bifurcated at 12–13 km depth from DHR has thrust up in the NW direction and reached at a shallower depth due to flexure bulging and anti-clockwise rotation of colliding Indian plate. An earthquake of M5.1 has also been located at the contact of fault F1 and DSR, which implies that this F1 fault is active and generates seismicity at the contact with DSR.

## Implications

**Figure 4:**
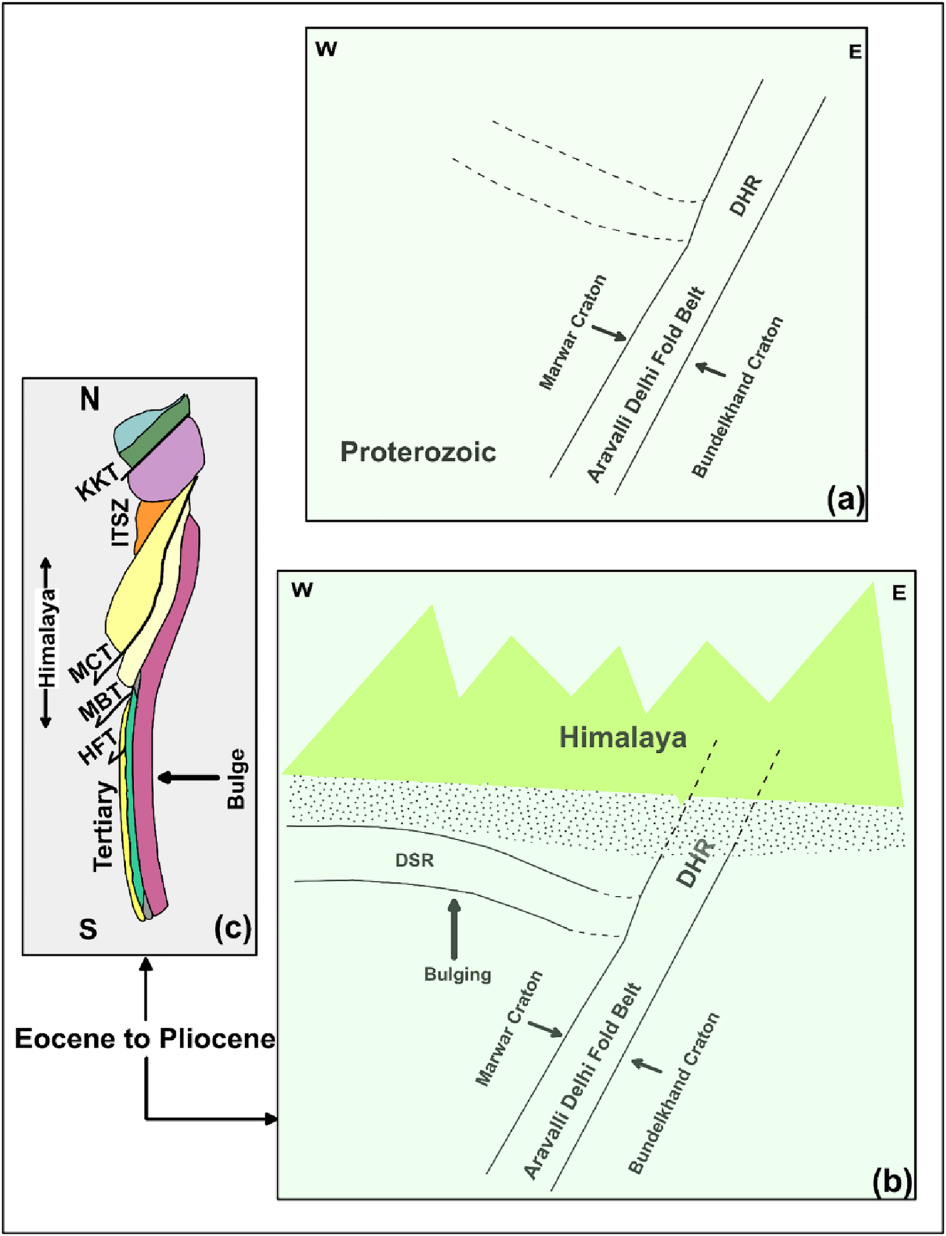
The schematic diagram (**a**) showing the formation of Aravallis during the Proterozoic due to the collision of Marwar Cratons and Bundelkhand Cratons, (**b**) the bulging in the forefront due to the collision of Indian and Eurasian plates during Eocene to Pliocene and (**c**) the N-S geological section of the Himalaya(after Irfan et al.^16^) showing the major thrusts (KKT: Karakoram Thrust; MCT: Main Central Thrust; MBT Main Boundary Thrust and HFT: Himalayan Frontal Thrust), suture zone (ITSZ: Indo Tsangpo Suture Zone) and formation of the bulge during Eocene to Pliocene.

The Himalayan Foreland Basin is a peripheral foreland basin, formed along the outer arc of the Himalayan orogen, on the subducting Indian Plate as a result of a collision with the Eurasian plate which was initiated in ~ 50 Ma in the Eocene. Foreland basins formed as over-riding plates when up-thrusted during the subduction of a denser slab creating shortening and crustal thickening, resulting in the creation of a mountain belt, and bending of the lithosphere. The bending of the lithosphere is known as lithospheric flexure. The present study infers that the DHR (which is the northern extension of DAFB formed due to the collision of the Bundelkhand Craton and Marwad Craton in the Proterozoic era (Fig. 4a)) bifurcated in the NW direction at a depth of 12–13 km. During the collisional tectonism (started at about 50 Ma) the DSR emerged by flexural bulging in the Himalayan foreland basin (Fig. 4b and c) with exposure of Proterozoic rocks at Tosham (~ 120 km WNW of Delhi in Haryana and Sargodha- Lahore (~ 600 km NW of Delhi). Both the DHR and DSR have shown gravity high indicating the high density of lower crustal rocks (mantle-activated products). A comparatively low resistivity (of 100 Ohm m) both at DHR and DSR might be due to the presence of folding and fracturing in the Delhi Aravalli supergroup and the presence of the fluid. Gokarn et al.^50^ have acquired MT sites in the NW of the current profile proposed a conductive zone at a depth of ~ 15 km, and confirmed its NW turning near Jind, Haryana. The results of Gokarn et al. 50 also support the findings and reasoning of the present study.

## Conclusion

A magnetotelluric investigation has been conducted for the first time at the seismically active proposed junction of DHR and DSR. The following observations are drawn from the investigation:

1. The Delhi Hardwar Ridge (DHR) and Delhi Sargodha Ridge (DSR) are the northward and northwestward extensions of the Proterozoic Delhi Aravalli Fold Belt, which are for the first time characterized by equal values of moderate resistivity of 100 Ohm m at two depths. The resistivity variation for DHR corresponds to 100 Ohm m from the surface to the depth of 20 km, whilst DSR is found associated with the same value of resistivity extending in the NW direction.
2. The gravity highs along DHR and DSR might be caused by the presence of metavolcanic and metasediments and the moderate resistivity is caused by the presence of fluid formed due to the metamorphism of subducted high conductivity and low-density rocks.
3. The DHR has been found striking NE-SW with a very shallow central axis (less than 400 m) having a width of 12–15 km forming half grabens on both limbs supported by shallow faults.
4. The DSR has been found bifurcated from DHR at a depth of 12–13 km and extended in the NW direction. The DSR has been generated due to flexure bulging in the Himalayan foreland basin.
5. A NE striking steep dipping reverse fault (F1) has also been identified about 15 km west of the DHR, the DSR which might be formed during the flexure bulging in the collisional tectonism.
6. The seismicity in the Rohtak and surroundings is located at the bifurcation points of DHR and DSR and at reverse fault F1.

## Methods

### Data acquisition and processing

The Magnetotelluric survey has been conducted along the NNW-SSE oriented profile of 08 sites starting from Suneria village in the NNW to the Mundhela village in the SSE (Fig. 2). The MT data recording framework ADU-07e of M/s Metromix, Gmbh, Germany utilized in obtaining MT data (five components: 2 electric (Ex and Ey) and 3 magnetic (Hx, Hy, and Hz) at all sites for a recording time of 72–96 h. The magnetic field recordings were made by utilizing MSF-06e coils, and the electric field estimations were made by utilizing Pb-PbCl_2_ electrodes. The dipole of 80–100 m was fixed for the electric field measurements at each site. The induction coils were buried approximately 1–1.5 ft and electrodes at the dipole ends were buried approximately 2 ft. The MT data is recorded at four recording frequencies; 16 kHz for 30 s, 8 kHz for 5 min, 4 kHz for 30 min, and 128 Hz for 72–96 h.

MT data are recorded as a time series. Before applying to the inversion algorithms, the data needs to be processed, including the removal of trends, data spikes, frequency domain conversion, and estimation of the transfer function. The MT time series processing software MAPROS by M/s Metromix, Gmbh, Germany, has been used to process the recorded time series. The recorded time series raw data of each magnetic and electric field have been manually inspected for marking jumps and spikes for exclusion while processing further. The trends were removed before converting it to the frequency domain. The data processing was rigorous and time-consuming due to the high anthropogenic noise in the study area. The resistivity curves of representative three sites are given in Supplementary Fig. 1. It is pertinent to mention that the data was acquired in the vicinity of populated habitats with heavy electricity and telecommunication networks, and a high cultural noise was observed.

### Dimensionality and directionality

The dimensionality examination is an important process for estimating the dimensions of the subsurface structure (1D, 2D, or 3D) in the study area. Swift’s skew (SS) is one of the parameters that specify the dimensionality of the subsurface structure^51^. The value of Swift’s skew ranges from 0 to 0.5^52^. A value < 0.2 designates the 1-D & 2-D subsurface structures^51^. The high skew values at a site designate a complex subsurface structure. In the present study, the SS value of ≤ 0.2 is observed (Supplementary Fig. 2a) at most of the sites suggesting the presence of a 1D/2D structure at the site.

Bahr’s skew (BS) is another parameter to propose dimensionality. It uses the impedance phase to measure the local 3-D distortion of regional 2-D fields rather than the impedance magnitudes^52^. Data is considered as 3D if it gives a BS value > 0.3^53^. In the current study, at most of the sites, a BS value of < 0.3 is observed at different periods (Supplementary Fig. 2b), indicating that MT response at the majority of periods indicates 2D dimensionality of regional geoelectrical structure. Site-wise average strike obtained after phase tensor analysis indicates the geoelectric strike is N37.5 E (Supplementary Fig. 2c). The ambiguity of 90 degrees in the geoelectric strike is resolved by observing the orientation of major structural features like DHR. The MT tensor is then decomposed in the regional coordinate frame using strike code^54^. MT tensors obtained in the regional coordinate frame are modeled for subsurface resistivity distribution.

### Data modelling

After careful processing, the decomposed and rotated apparent resistivity and phase data from a period of 0.001 to 10 s of all 08 sites are subjected to the 2D inversion using the robust code proposed by Rodi and Mackie^55^, which is also readily available with the MT data modeling software. This inversion proposes the regularized solutions named Tikhonov Regularization following the method of non-linear conjugate gradients (NLCG). Finite-difference equations were used to calculate forward model simulations based on Maxwell's equations analogous to networks. The smoothing operator/regularization parameter ζ, which is a measure of compromise between the model smoothness and data fit, is a significant parameter in the NLCG scheme. In the present case, the L-curve criteria^56,57^ were utilized to ascertain the ζ value. Profile error floors have been set at 30 percent for apparent resistivity and 1.5° for the phase. The inversion was initiated on a scant grid and later on, new rows/columns were included where important structures were detected. A mesh size of 41 × 90 found optimal. The inversion was started at a uniform half-space with a resistivity of 100 Ohm·m. It took 100 iterations to attain a pre-set root mean square error (RMS) level, which is defined as the RMS of the sum of a misfit in rho and phase of TM and TE modes. The 2D resistivity depth sections up to a depth of 20 km are shown in Fig. 3 for the TM mode and Supplementary Figs. 3 and 4 for the TE and TE + TM modes, respectively. The computed pseudo sections for the TM, TE, and TE + TM mode responses are given in Supplementary Figs. 6, 7, and 8, respectively. A similarity in resistivity distribution can be seen between computed and observed pseudo sections in Supplementary Figs. 6, 7 and 8. The fit with the data at three sites is given in Supplementary Fig. 9. An RMS error of 3.8 is estimated.

### Supplementary Information

Supplementary Information.

## Data Availability

The datasets generated during and/or analyzed during the current study will be made available at a reasonable request to the Director, NCS (director-ncs@gov.in).
