# Supplementary material for: Geoelectric characterisation of the junction of seismically active Delhi Hardwar Ridge and Delhi Sargodha Ridge
Source: Sci Rep. 2023 Oct 28;13:18488. doi: 10.1038/s41598-023-42722-w (PMC10613208; doi:10.1038/s41598-023-42722-w)
Supplement: Supplementary file 1 — Supplementary Information. [file 41598_2023_42722_MOESM1_ESM.docx]

**Geoelectric Characterisation of the Junction of seismically active Delhi Hardwar Ridge and Delhi Sargoda Ridge**

Gautam Rawat^1^_,_ Kapil Mohan^2*^, S. Dhamodharan^1^, Harendra Dadhich^2^, Prasanta Chingtham^2^, Kalachand Sain^1^ and O.P. Mishra^2^

**^1^**Wadia Institute of Himalayan Geology, 33 GMS Road, Dehradun 248 001, India, India

**^2^**National Center for Seismology, Ministry of Earth Sciences, Lodhi Road, New Delhi-110003

**email:** kapil.mohan12@gov.in

| 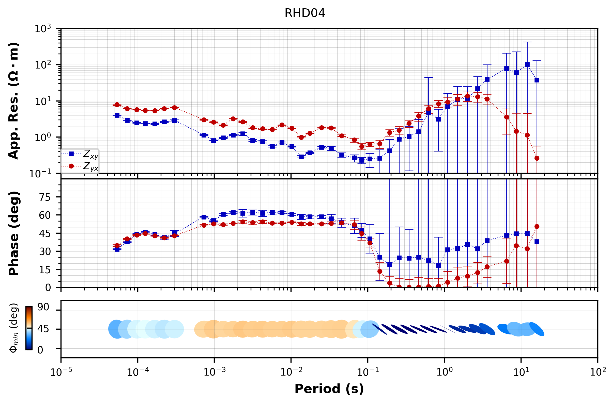**Site 4** |
| --- |
| 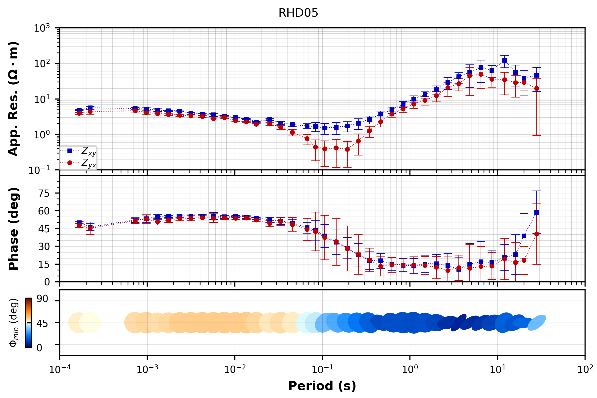**Site 5** |
| 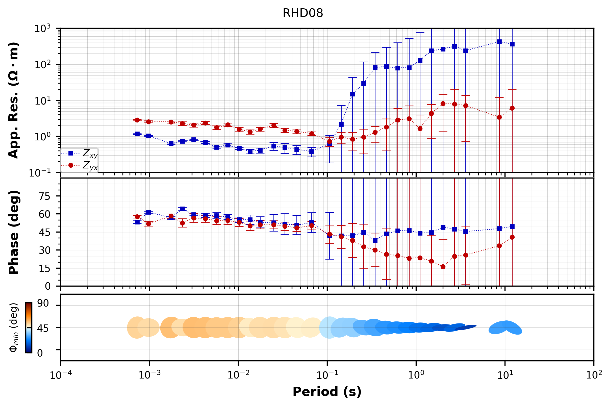**Site 8** |

**Supplementary Figure 1:** Apparent resistivity curves and phase ellipses for the MT site No. 4,5 and 8.


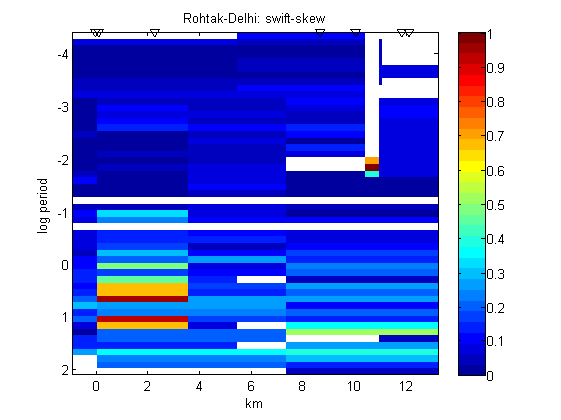


**(a)**


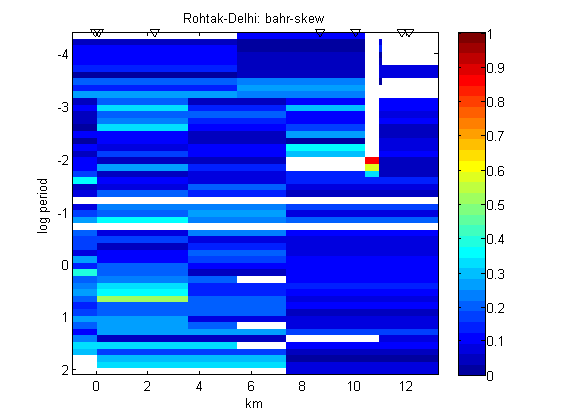


**(b)**

**
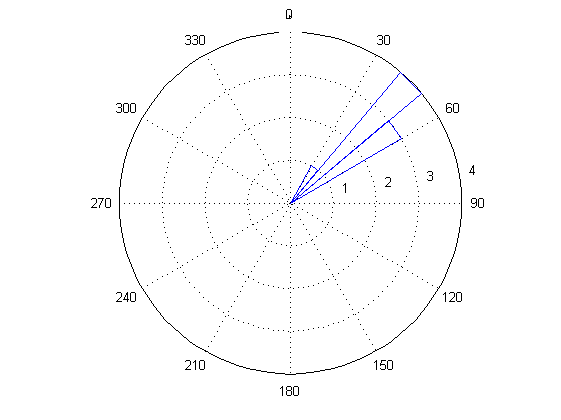
**

**(c)**

**Supplementary Figure 2:** (a) Swift’s, (b) Bahr’s skew values at different periods at all sites along the profile. Most of the period range is showing a 2D structure in the area and (c). Rose diagram of estimated strike values estimated after phase tensor analysis.

**
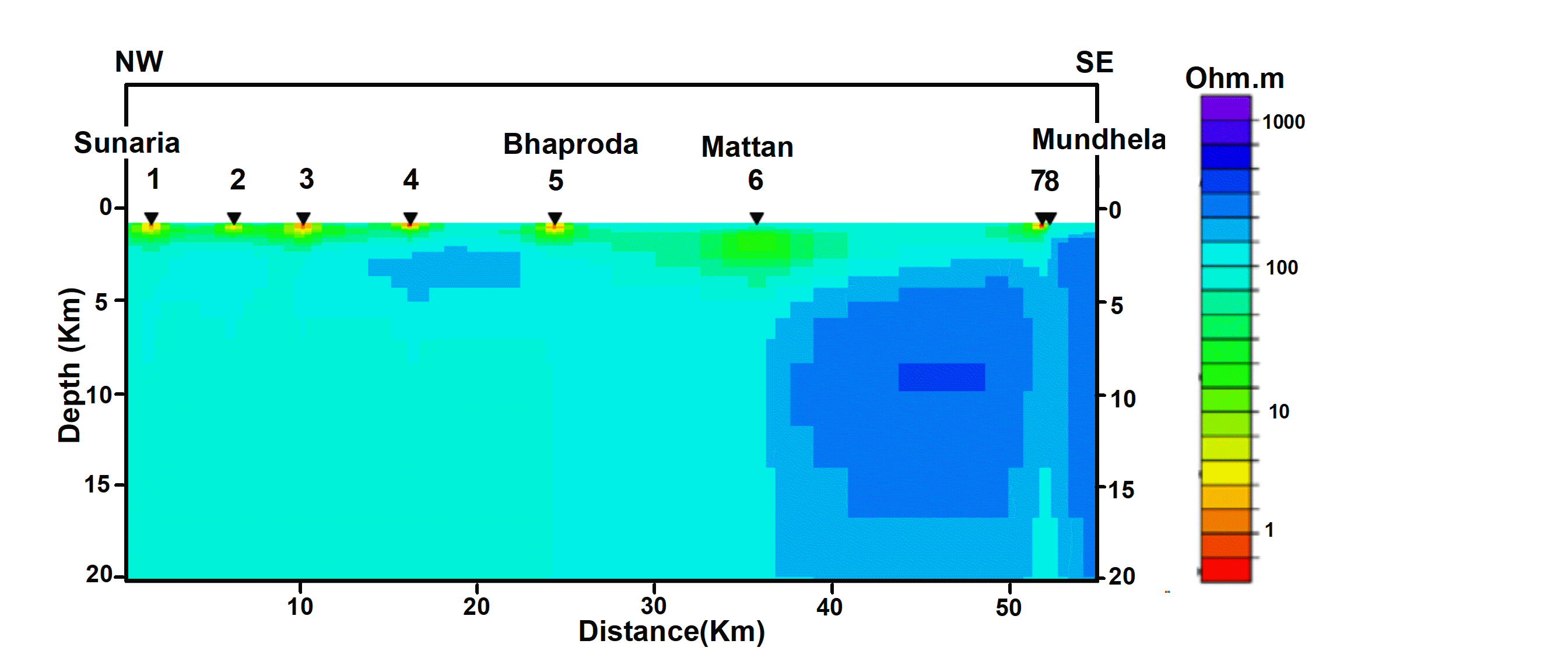
**

**Supplementary Figure 3:** The 2D resistivity depth section produced from the TE mode inversion.

**
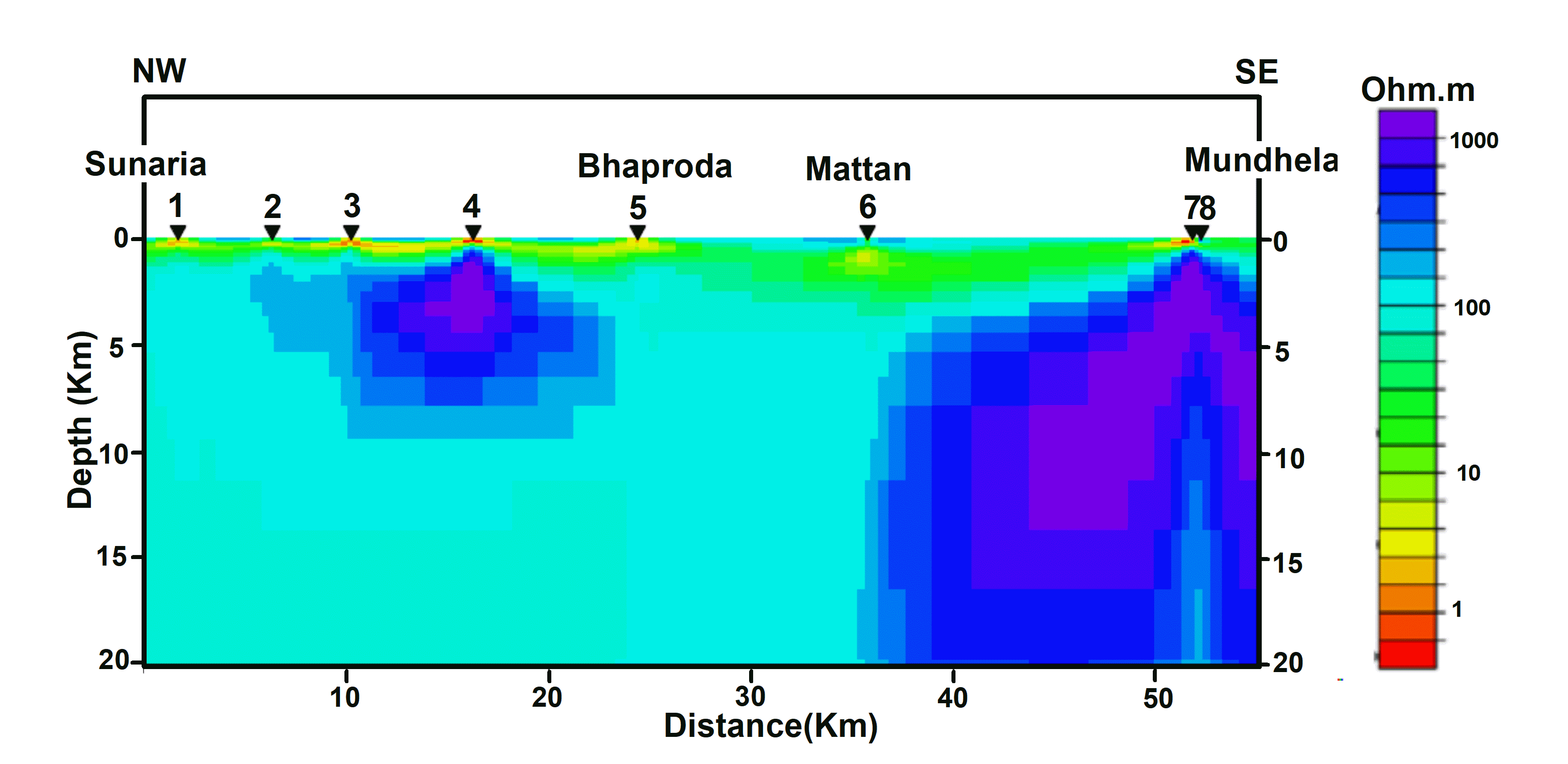
**

**Supplementary Figure 4:** The 2D resistivity depth section produced from the TE+TM mode inversion.


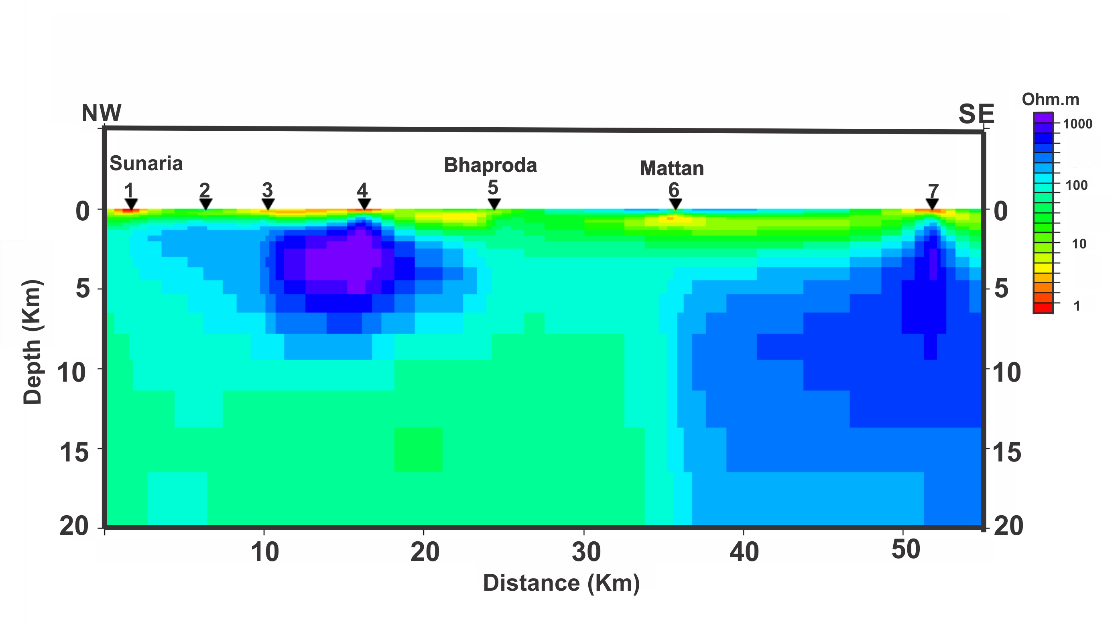


**Supplementary Figure 5:** The 2D resistivity depth section produced from the TM mode inversion removing site 8.


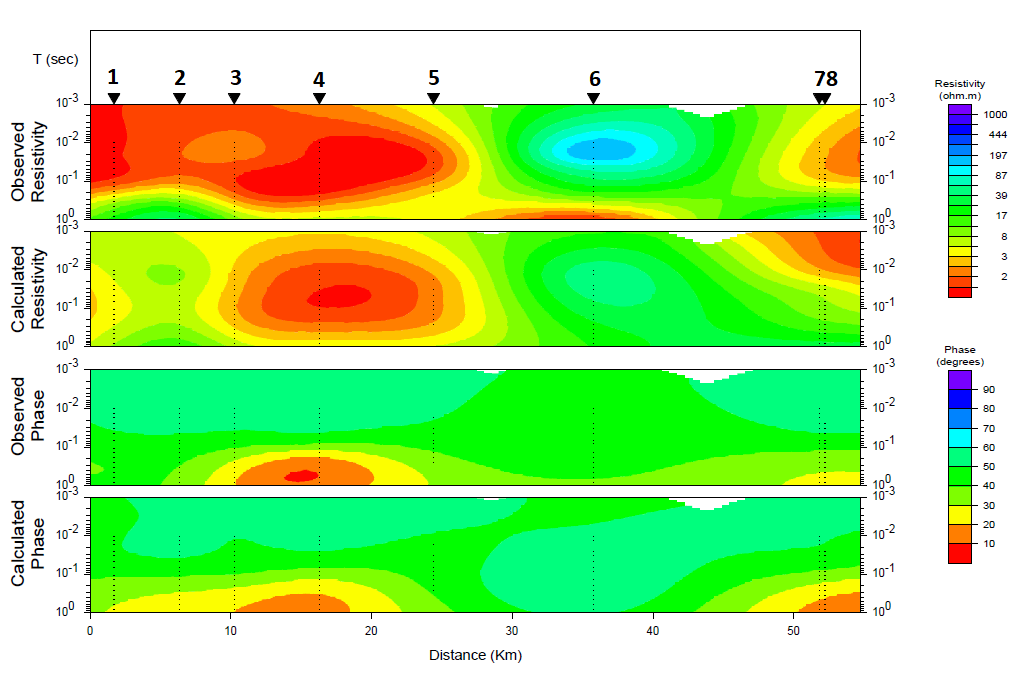


**Supplementary Figure 6:** The TM mode Pseudo-sections of the observed and modeled apparent resistivity and phase values.


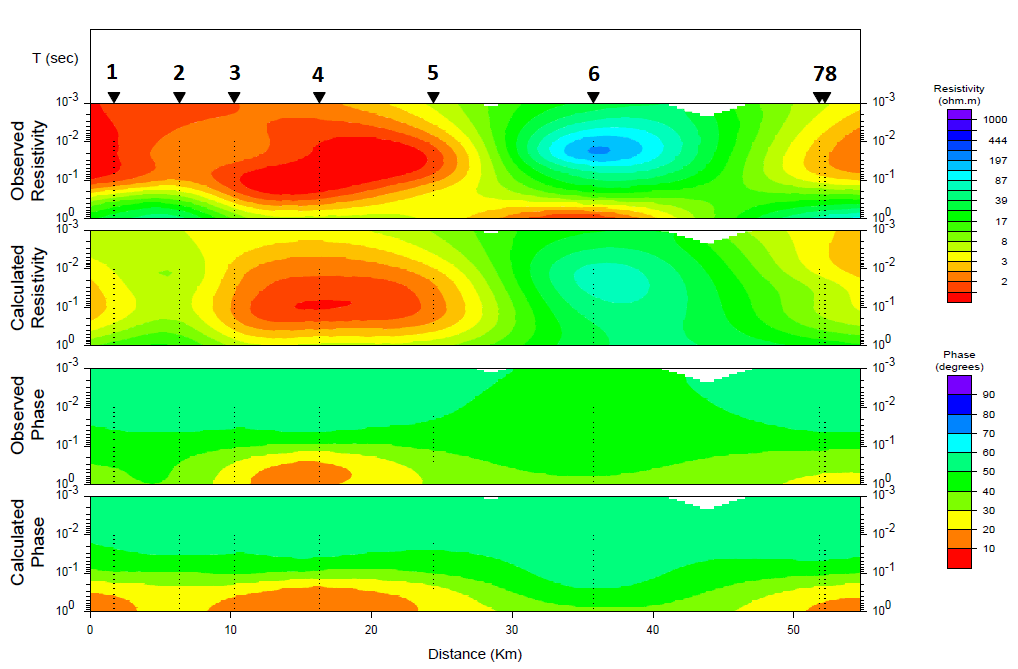


**Supplementary Figure 7:** The TE mode Pseudo-sections of the observed and modeled apparent resistivity and phase values.

**
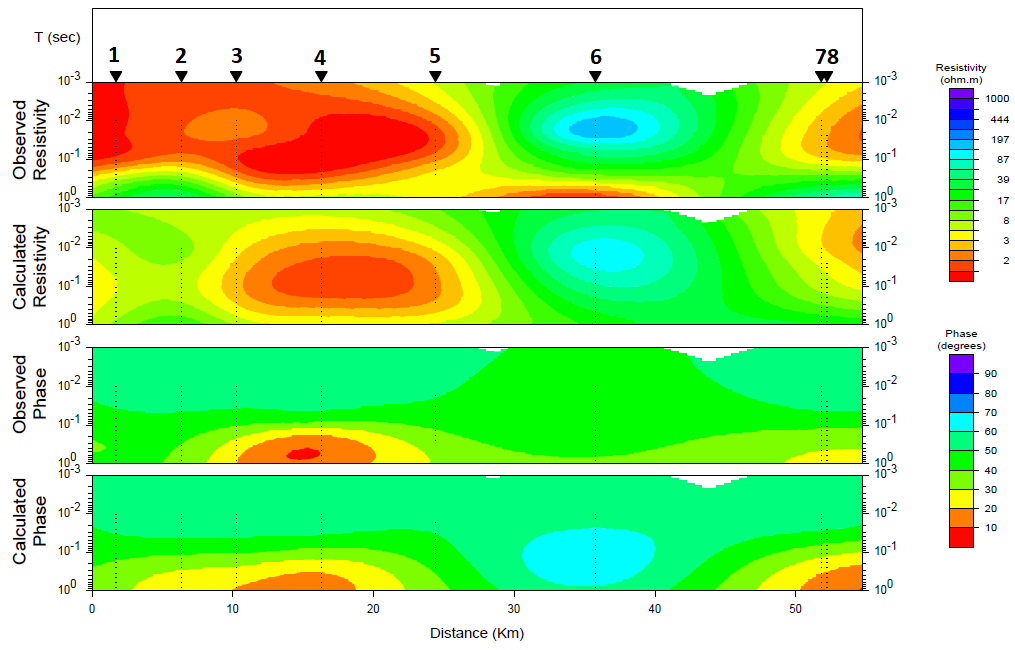
**

**Supplementary Figure 8:** The TE+TM mode Pseudo-sections of the observed and modeled apparent resistivity and phase values.


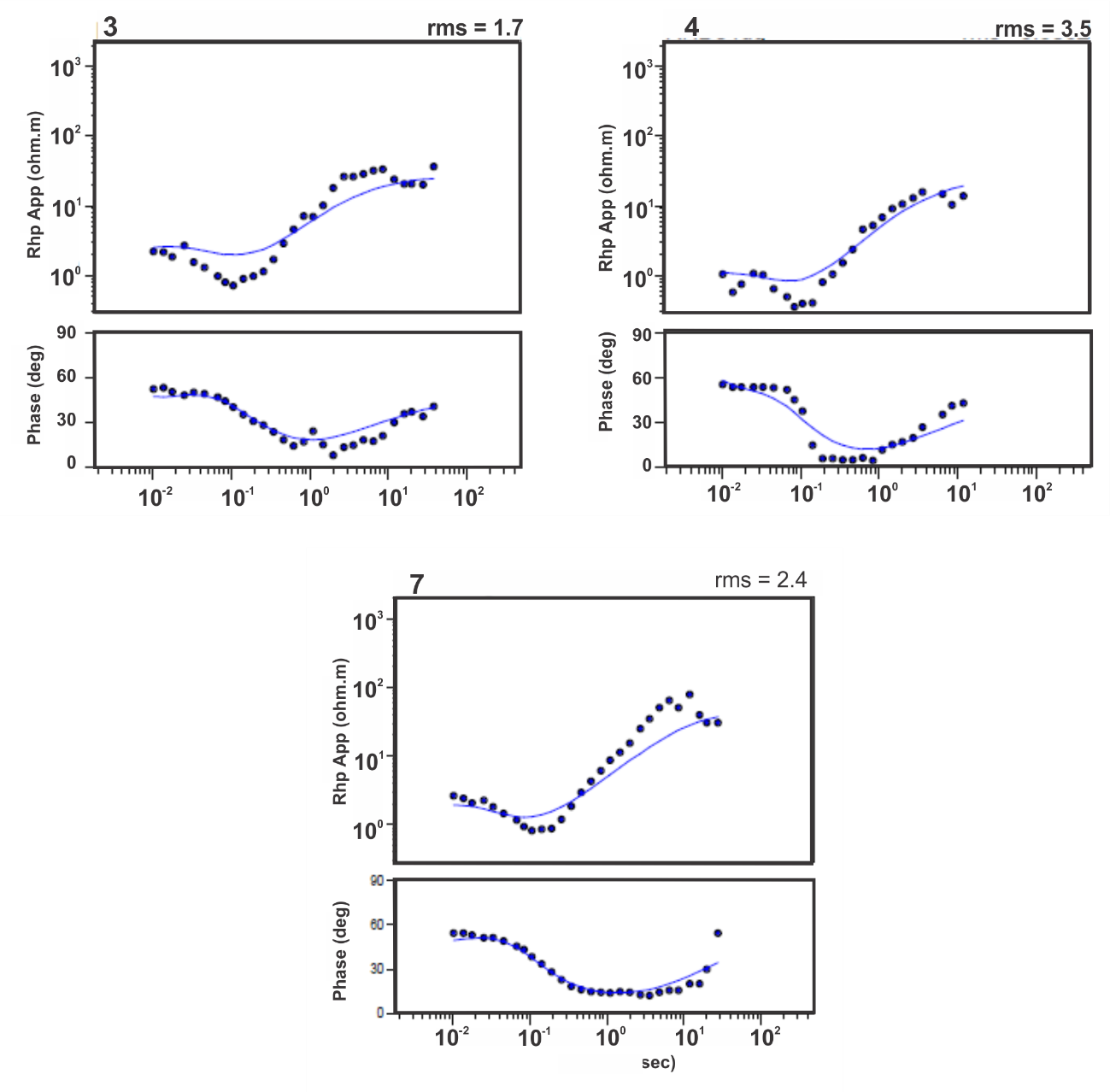


**Supplementary Figure 9:** The TM mode observed and modeled apparent resistivity and phase values/ fit with the data.
